# Supplementary material for: Educational Intervention to Decrease Justification of Adolescent Dating Violence: A Comparative Quasi-Experimental Study
Source: Healthcare (Basel). 2023 Apr 18;11(8):1156. doi: 10.3390/healthcare11081156 (PMC10138385; doi:10.3390/healthcare11081156)

SUPPLEMENTARY MATERIAL

Figure S1. The extent to which all the participants justify physical violence as a means of conflict resolution, based on the AADS test.

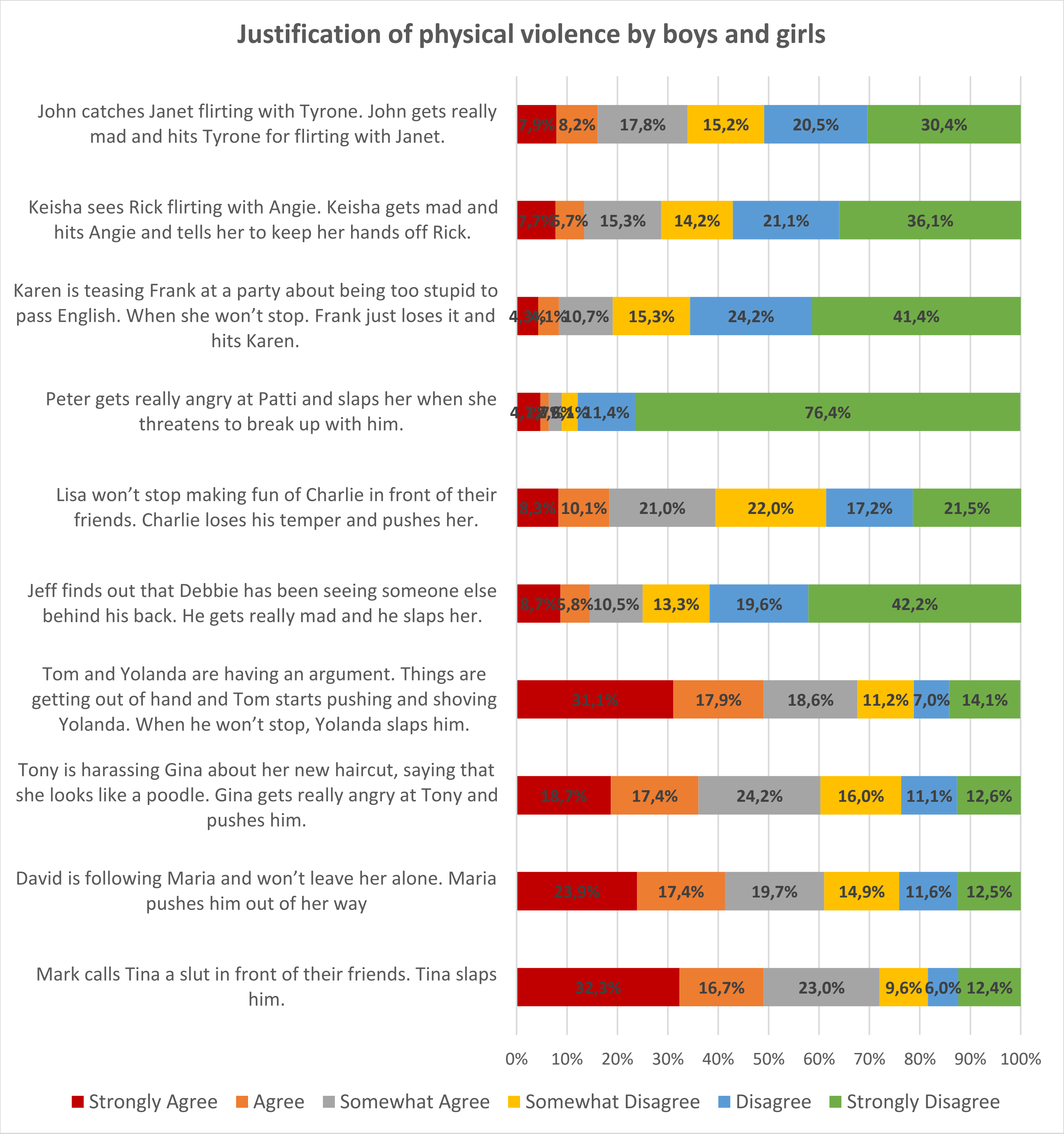

Table S1. The extent to which girls justify female psychological violence against partners, based on the JVCT test.

	Level of justification				
	**Never**	**In extreme situations**	**Rarely**	**In some cases**	**In many cases**
Insulting or threatening the boyfriend/girlfriend.	*76,8%*	*12,1%*	*4,3%*	*4,3%*	*2,5%*
Being grumpy when talking about a subject.	*16,0%*	*21,3%*	*30,5%*	*22,8%*	*9,4%*
Suddenly leaving the room, house or place of the discussion	*22,0%*	*26,5%*	*21,7%*	*14,1%*	*15,7%*
Doing or saying something to upset the boyfriend/girlfriend.	*65,5%*	*12,9%*	*11,2%*	*6,9%*	*3,6%*
Do not let the boyfriend/girlfriend see or talk to your family.	*87,4%*	*4,8%*	*4,0%*	*2,0%*	*1,8%*
Turn the boyfriend/girlfriend's family or friends against him/her.	*89,4%*	*4,8%*	*2,8%*	*1,3%*	*1,8%*
Don't let your boyfriend/girlfriend do things to be with you.	*70,0%*	*13,2%*	*8,4%*	*4,8%*	*3,6%*
Interfering in the relationship with family members.	*76,0%*	*13,0%*	*4,3%*	*3,6%*	*3,1%*
Being jealous of their friends and suspicious of them.	*40,1%*	*28,9%*	*16,8%*	*7,9%*	*6,3%*
Becoming jealous of other boys/girls	*32,0%*	*26,1%*	*18,3%*	*12,7%*	*10,9%*
Check what he/she does and demand to know where he/she has been.	*75,9%*	*10,7%*	*4,1%*	*5,6%*	*3,8%*
Accusing the boyfriend/girlfriend of going out with another boy/girlfriend.	*57,1%*	*24,1%*	*8,6%*	*5,1%*	*5,1%*

Table S2. The extent to which boys justify female psychological violence against partners, based on the JVCT test.

	Level of justification				
	**Never**	**In extreme situations**	**Rarely**	**In some cases**	**In many cases**
Insulting or threatening the boyfriend/girlfriend.	*74,2%*	*12,9%*	*7,7%*	*2,6%*	*2,6%*
Being grumpy when talking about a subject.	*21,9%*	*17,1%*	*24,5%*	*23,3%*	*13,3%*
Suddenly leaving the room, house or place of the discussion	*22,4%*	*24,7%*	*21,4%*	*18,4%*	*13,2%*
Doing or saying something to upset the boyfriend/girlfriend.	*61,8%*	*16,4%*	*11,2%*	*5,4%*	*5,2%*
Do not let the boyfriend/girlfriend see or talk to your family.	*85,2%*	*5,6%*	*3,7%*	*1,6%*	*3,7%*
Turn the boyfriend/girlfriend's family or friends against him/her.	*82,2%*	*9,9%*	*3,3%*	*1,6%*	*3,1%*
Don't let your boyfriend/girlfriend do things to be with you.	*63,7%*	*17,5%*	*10,6%*	*3,5%*	*4,7%*
Interfering in the relationship with family members.	*70,1%*	*14,6%*	*8,9%*	*2,8%*	*3,5%*
Being jealous of their friends and suspicious of them.	*43,8%*	*26,9%*	*13,1%*	*8,9%*	*7,3%*
Becoming jealous of other boys/girls	*35,0%*	*24,2%*	*19,0%*	*12,4%*	*9,4%*
Check what he/she does and demand to know where he/she has been.	*67,1%*	*15,3%*	*7,3%*	*6,8%*	*3,5%*
Accusing the boyfriend/girlfriend of going out with another boy/girlfriend.	*52,2%*	*23,8%*	*11,8%*	*5,9%*	*6,4%*

Table S3. The extent to which girls justify male psychological violence against partners, based on the JVCT test.

	Level of justification				
	**Never**	**In extreme situations**	**Rarely**	**In some cases**	**In many cases**
Insulting or threatening the boyfriend/girlfriend.	79,2%	9,9%	4,2%	3,5%	3,2%
Being grumpy when talking about a subject.	15,7%	22,1%	28,9%	23,4%	9,9%
Suddenly leaving the room, house or place of the discussion	20,9%	27,7%	22,9%	13,0%	15,5%
Doing or saying something to upset the boyfriend/girlfriend.	66,3%	13,3%	10,5%	6,4%	3,6%
Do not let the boyfriend/girlfriend see or talk to your family.	86,5%	5,1%	3,8%	2,3%	2,3%
Turn the boyfriend/girlfriend's family or friends against him/her.	87,5%	4,6%	3,8%	2,3%	1,8%
Don't let your boyfriend/girlfriend do things to be with you.	69,8%	12,5%	8,2%	5,4%	4,1%
Interfering in the relationship with family members.	73,6%	13,1%	6,7%	3,1%	3,6%
Being jealous of their friends and suspicious of them.	40,1%	29,1%	15,8%	7,7%	7,4%
Becoming jealous of other boys/girls	30,4%	26,8%	18,6%	11,7%	12,5%
Check what he/she does and demand to know where he/she has been.	74,7%	9,0%	4,6%	5,6%	6,1%
Accusing the boyfriend/girlfriend of going out with another boy/girlfriend.	55,2%	24,3%	9,5%	5,6%	5,4%

Table S4. The extent to which boys justify male psychological violence against partners, based on the JVCT test.

	**Level of justification**				
	**Never**	**In extreme situations**	**Rarely**	**In some cases**	**In many cases**
Insulting or threatening the boyfriend/girlfriend.	75,0%	12,7%	5,6%	3,6%	3,1%
Being grumpy when talking about a subject.	23,6%	16,1%	26,4%	22,2%	11,7%
Suddenly leaving the room, house or place of the discussion	24,1%	23,7%	21,8%	19,0%	11,4%
Doing or saying something to upset the boyfriend/girlfriend.	62,8%	16,2%	10,9%	5,8%	4,4%
Do not let the boyfriend/girlfriend see or talk to your family.	83,4%	6,2%	5,1%	2,1%	3,2%
Turn the boyfriend/girlfriend's family or friends against him/her.	82,2%	9,2%	4,4%	1,6%	2,5%
Don't let your boyfriend/girlfriend do things to be with you.	64,4%	16,9%	11,1%	4,2%	3,5%
Interfering in the relationship with family members.	69,4%	14,1%	11,1%	3,0%	2,5%
Being jealous of their friends and suspicious of them.	44,0%	28,8%	12,9%	8,1%	6,2%
Becoming jealous of other boys/girls	35,3%	25,6%	19,1%	10,8%	9,2%
Check what he/she does and demand to know where he/she has been.	66,7%	14,3%	8,1%	6,9%	3,9%
Accusing the boyfriend/girlfriend of going out with another boy/girlfriend.	51,8%	24,2%	10,1%	7,4%	6,5%

Figure S2. Analysis of the changes on physical violence, based on the AADS dimensions, after the educational intervention. Statistical analyses were performed through a Wilcoxon test. Box plots depict 5th (lower edge) and 95th (upper edge) percentiles and median (horizontal bar).

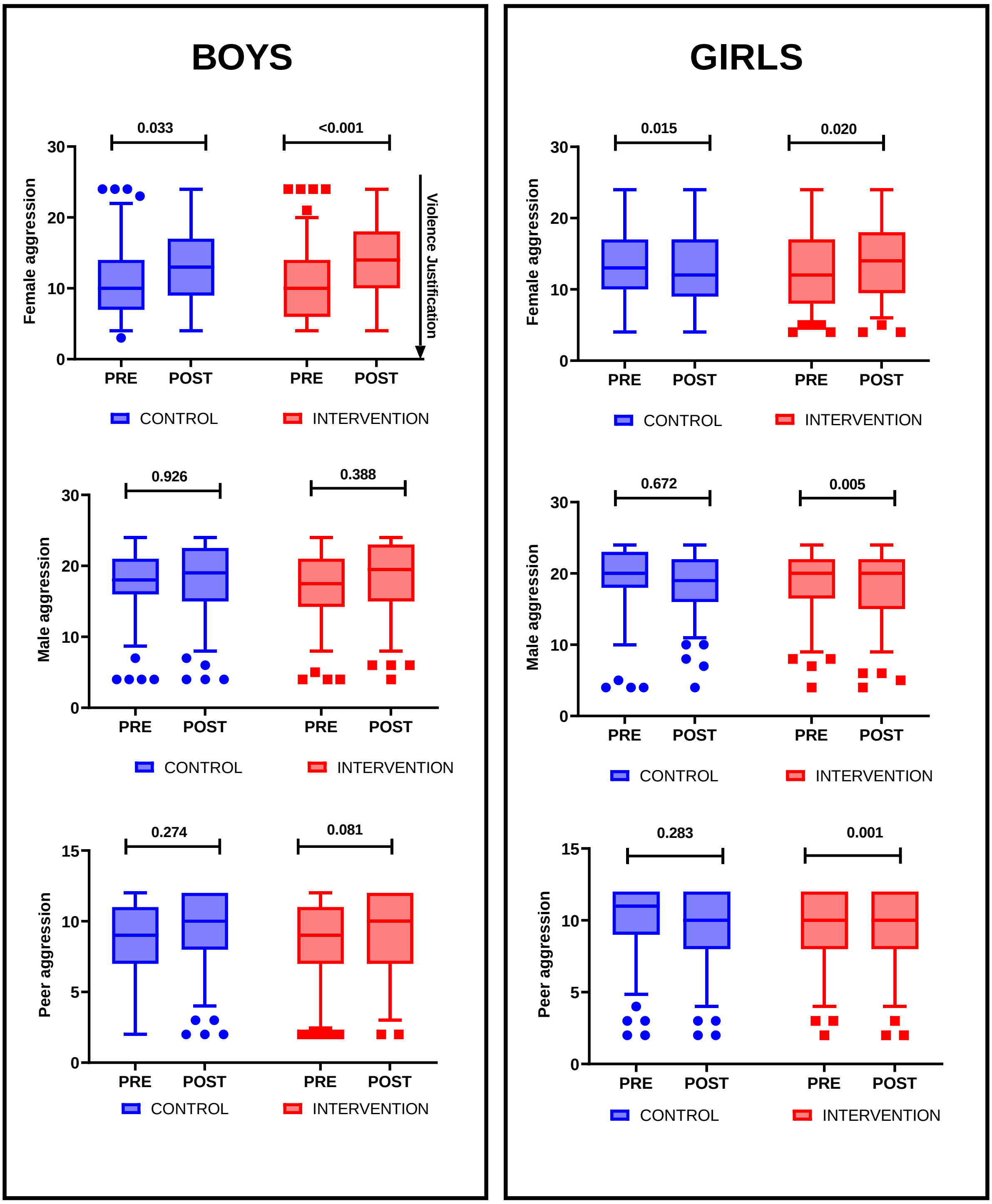

Figure S3. Analysis of the changes on psychological violence, based on the JVCT dimensions, after the educational intervention in the boys group. Statistical analyses were performed through a Wilcoxon test. Box plots depict 5th (lower edge) and 95th (upper edge) percentiles and median (horizontal bar).

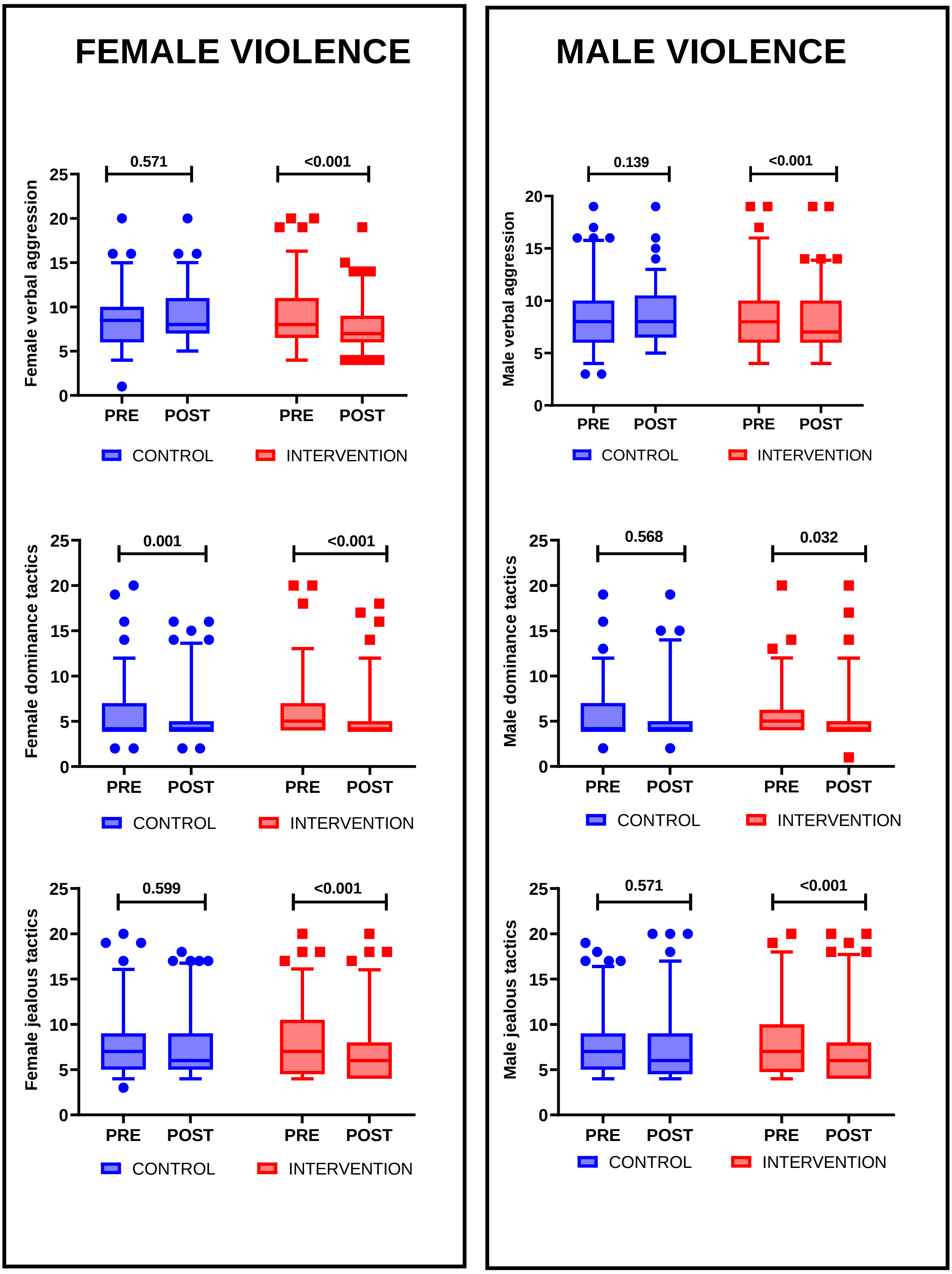

Figure S4. Analysis of the changes on psychological violence, based on the JVCT dimensions, after the educational intervention in the girls group. Statistical analyses were performed through a Wilcoxon test. Box plots depict 5th (lower edge) and 95th (upper edge) percentiles and median (horizontal bar).

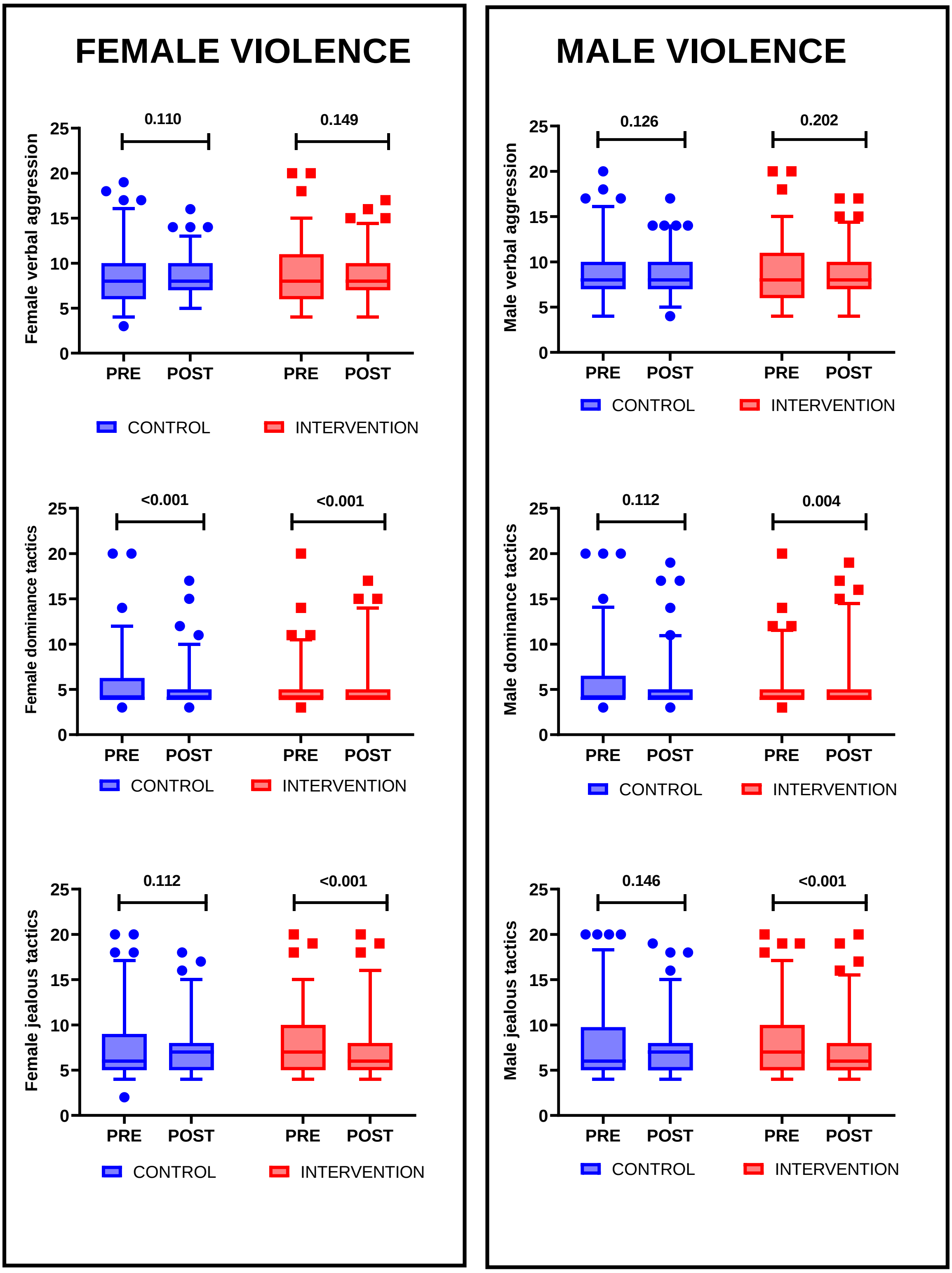

Supplement: Supplementary file 1 [file healthcare-11-01156-s001.zip › healthcare-2289014-supplementary.pdf]
